# Vacuolar compartments preserved among loosely packed amyloplasts account for heat-induced rice chalky formation under low nitrogen conditions

**DOI:** 10.1007/s00425-025-04793-z

**Published:** 2025-08-13

**Authors:** Yuto Hatakeyama, Kenichi Wakamatsu, Akio Tanaka, Taku Tanogashira, Hiroshi Nonami, Hiroshi Nakano, Hiroshi Wada

**Affiliations:** 1 Kyushu Okinawa Agricultural Research Center, National Agriculture and Food Research Organization, Chikugo, Fukuoka Japan; 2 Graduate School of Agriculture, Ehime University, Matsuyama, Ehime, Japan; 3 Kagoshima Prefectural Institute for Agricultural Development, Minamisatsuma, Kagoshima, Japan

**Keywords:** Amyloplast, Chalkiness, High temperature, Nitrogen, Organelle, *Oryza sativa* L., Protein body, Vacuole

## Abstract

**Main conclusion:**

The regulation of vacuolar compartmentation and protein synthesis during the early ripening stage might be responsible for rice appearance at high temperature.

**Abstract:**

High temperature at the early ripening stage disrupts protein synthesis to arrest starch and storage protein accumulation in the rice endosperms, leading to the occurrence of chalky kernels (CK), such as white-back kernels (WBK) and basal-white kernels (BWK). In contrast, adequate nitrogen (N) application might sustain protein synthesis and reduce chalky kernels. These processes might be associated with the regulation of vacuolar compartmentation and protein synthesis during heat adaptation, yet the exact cellular dynamics behind the reduction of endosperm air space when applying N have not been examined in the fields. In this study, plants at different N levels were treated under the same high temperatures in the fields and morphological analysis were carried out to examine the time course of changes in organelles compartmentation during the N-enhanced mitigation process. Light and transmission electron microscopic observations were conducted at dorsal and basal endosperm cells, at which chalky formation was presumed to occur under low N conditions during kernel development at high temperature. Results show that CK reached 25.0% in no N-applied plants under heat, whereas N treatment contrastingly decreased CK formation down to 10.7%. In the mature kernels, the areas of chalky cells, amyloplasts, and protein bodies (PBs) were smaller in chalky cells, compared with translucent cells. At the middle ripening stage, volumetric enlargement of protein storage vacuole concomitant with the arrested amyloplast development were both observed in the putative growing chalky cells, resulting in the formation of CK at the late ripening stage. In contrast, N application ameliorated the effect on rice appearance by diminishing the vacuolar size and enhancing protein synthesis to ensure cell size and amyloplast and PB development, increasing the transparency. Therefore, it is proposed that regulation of vacuolar compartmentation and protein synthesis at the ripening stage might be responsible for rice appearance under field conditions.

**Supplementary Information:**

The online version contains supplementary material available at 10.1007/s00425-025-04793-z.

## Introduction

High-temperature conditions have been frequently increasing the occurrence of rice chalkiness (Jagadish et al. 2015; Morita et al. 2016; Wada 2019; Xiong et al. 2017), which is currently a serious concern in rice production under climate change. The chalky appearance is ascribed to morphological traits of the endosperm cells; the formation of numerous air spaces among the amyloplasts and protein bodies (PBs) leads to random light reflection (Tashiro and Wardlaw 1991). Chalky kernel (CK) begins to occur typically at 24 °C on average during 20 days after heading (DAH), with a slope of about 10% per 1 °C from 28 to 29 °C in a leading cultivar, Koshihikari (Morita et al. 2016). It is known that there are several types of CKs based on the within-kernel position of chalky formation, and each environmental condition, such as heat, low light intensity, and foehn-induced dry wind conditions, cause different types of chalky rice (Tashiro and Wardlaw 1991; Morita et al. 2016). White-back kernels (WBK) that exhibit chalkiness in the part of the outer endosperm (OE) regions longitudinally aligned along the dorsal side of the kernels, are frequently observed when the air temperature during the early ripening stage is high (Kobayashi et al. 2007; Morita et al. 2016). Another type of CK, called ‘basal-white rice (BWK)’ exhibits chalkiness in the basal endosperms adjacent to the embryo. BWK is also induced by high temperatures. It has been empirically known in the fields that supplying adequate nitrogen (N) as a topdressing alleviate the occurrence of these WBK and BWK even under heat conditions (Wakamatsu et al. 2008; Morita et al. 2016; Tsukaguchi et al. 2016; Dou et al. 2017) and the countermeasures regarding the optimal N application under heat conditions have been developed (e.g., Nakano et al. 2022), although the underlying mechanisms at the cell level remain obscure.

To date, it has been reported that the down-regulation of some starch synthesis-related genes (Yamakawa et al. 2007; Hakata et al. 2012), up-regulation of starch-degrading α-amylase-encoding genes (Yamakawa et al. 2007; Hakata et al. 2012) and increasing in excessive reactive oxygen species (ROS) (Shiraya et al. 2015; Suriyasak et al. 2017) might be caused under the high temperature conditions. Most studies on rice chalky formation have been conducted on whole kernels under heat stress conditions. However, there are little attempts in which the above-mentioned N-applied effects have been considered together with the two temperature treatments. Furthermore, the chalky area is confined to a part of the kernel, showing cell heterogeneity. Conducting cell-specific approaches confined to the putative chalky zone would be effective to better understand the underlying mechanisms of chalky formation and N-induced alleviation effect.

At maturation, rice endosperms are typically composed of numerous starch granules and storage proteins, and the ratio of the storage proteins corresponds to 5–8% of the rice endosperm (Hoshikawa 1989). Storage proteins are stored into two types of PBs, protein body type I (PBI) formed by accumulating prolamin in the rough endoplasmic reticulum, and protein body type II (PBII) originated from protein storage vacuole (PSV) with the accumulation of glutelin precursors (pro-glutelin), acidic (α)-glutelin, α-globulin, and basic (β)-glutelin in (Yamagata et al. 1982; Onda et al. 2011). Furthermore, PBI has two types of layers identifiable under transmission electron microscopy (TEM), namely an inner layer of cysteine-rich 10-kDa prolamins (CysR10P) that is rich in disulfide bonds, and an outer layer containing a mixture of other prolamins (Onda et al. 2011; Nagamine et al. 2011; Saito et al. 2012; Wada et al. 2019). There is growing evidence to suggest that the regulation of protein synthesis plays a key role in the formation of heat-induced rice chalkiness (Kawakatsu et al. 2010; Tang et al. 2018; Wada et al. 2019; Ishimaru et al. 2020). Recently, on-site single-cell metabolome analysis, termed ‘picolitre pressure-probe electrospray-ionization mass spectrometry (picoPPESI-MS)’, combined with TEM observation has demonstrated that heat-induced chalky formation (i.e., WBK) might be caused by the preservation of vacuole-like structure in the endosperm cells with inadequate accumulation of amyloplasts and PBs, namely PBII (Wada et al. 2019). Regarding the formation of foehn-induced ring-shaped chalky rice, generally known as milky-white rice, the preservation of vacuole-like structures occurred as a consequence of osmotic adjustment in the inner endosperm cells was shown the be the main cause (Hatakeyama et al. 2018). The actual gap space in the chalky zone appears to be attributed to the loss of endosperm transparency; however, little has been considered about the organelle rearrangement in the past. It is important to keep in mind that chalkiness generally appears when the spatial ratio of the gap space observed in the zone was > 10% of the chalky cells (Wada et al. 2014; Hatakeyama et al. 2018). This suggests that investigating the spatial rearrangement of organelle compartmentation throughout ripening stages might be effective for understanding the mechanisms of heat-induced chalky formation and the N-applied alleviate effects. However, most experiments conducted at the cell level were confined to growth chamber experiments (e.g., Wada et al. 2019). To our knowledge, no attempt has been made in field-grown rice plants for testing how N application affects the compartmentation at the cell level.

In this study, we have hypothesized that the preservation of vacuoles in the cells concomitant with the disruption of protein biosynthesis might be a direct cause of heat-induced chalky formation and that N application might enhance protein synthesis to improve the kernel quality in field-grown rice plants exposed to heat conditions. Therefore, the pattern of endosperm organelle arrangement with amyloplasts, PBs, and vacuoles in both dorsal and basal endosperm cells in relation to N availability was tested during the ripening stage using a light microscope and TEM.

## Materials and methods

### Field experiments

Rice plants (*Oryza sativa* L. cv. Koshihikari) were grown in 2017 and 2018 on Lowland Paddy soil in the field at the Kagoshima Prefectural Institute for Agricultural Development, Minamisatsuma, Kagoshima, Japan (31°28’ N, 130°20’ E; 25 m above sea level). Seedlings grown in a greenhouse for 21 and 23 d in 2017 and 2018, respectively, were transplanted by a rice planting machine in the irrigated field on 19 May, 2017 and 16 May, 2018, respectively. Planting densities in 2017 and 2018 were 21.6 and 20.1 hills per square meter, respectively (hill spacing of 0.2 × 0.15 m in 2017, and of 0.2 × 0.17 m in 2018). As a basal dressing, 4 g N per square meter with 6.0 g phosphorus (P) m^−2^, 4.7 g potassium (K) m^−2^ in the form of chemical fertilizer was broadcasted by hand prior to the transplanting.

Three treatments based on the applied amount and timing of N application were designed as follows: (i) only basal dressing without topdressing (0 N treatment); (ii) the basal dressing with topdressing containing 2.0 g N m^−2^ at 20 d before heading (2 N treatment); and (iii) the basal dressing with two times topdressing containing 4.0 g N m^−2^ as a total amount at 20 d and 10 d before heading (4 N treatment). For 2 N and 4 N treatments, plants at 20 d and 10 d before heading received 2.0 g N m^−2^ and 2.0 g K m^−2^ and 2.0 g N m^−2^, 3.0 g P m^−2^, 2.3 g K m^−2^, respectively, in the form of ammonium sulfate. These treatments were arranged in a randomized complete block design with three replications. The experimental plots in 2017 and 2018 were 7.3 and 4.0 m^2^, respectively (2.6 m-wide × 2.8 m-long in 2017, and 2.0 m-wide × 2.0 m-long in 2018). Heading dates in 2017 and 2018 were 17 July and 15 July, respectively. The field was evenly irrigated to continuously maintain above the soil.

### Microscopy

**Fig. 1:**
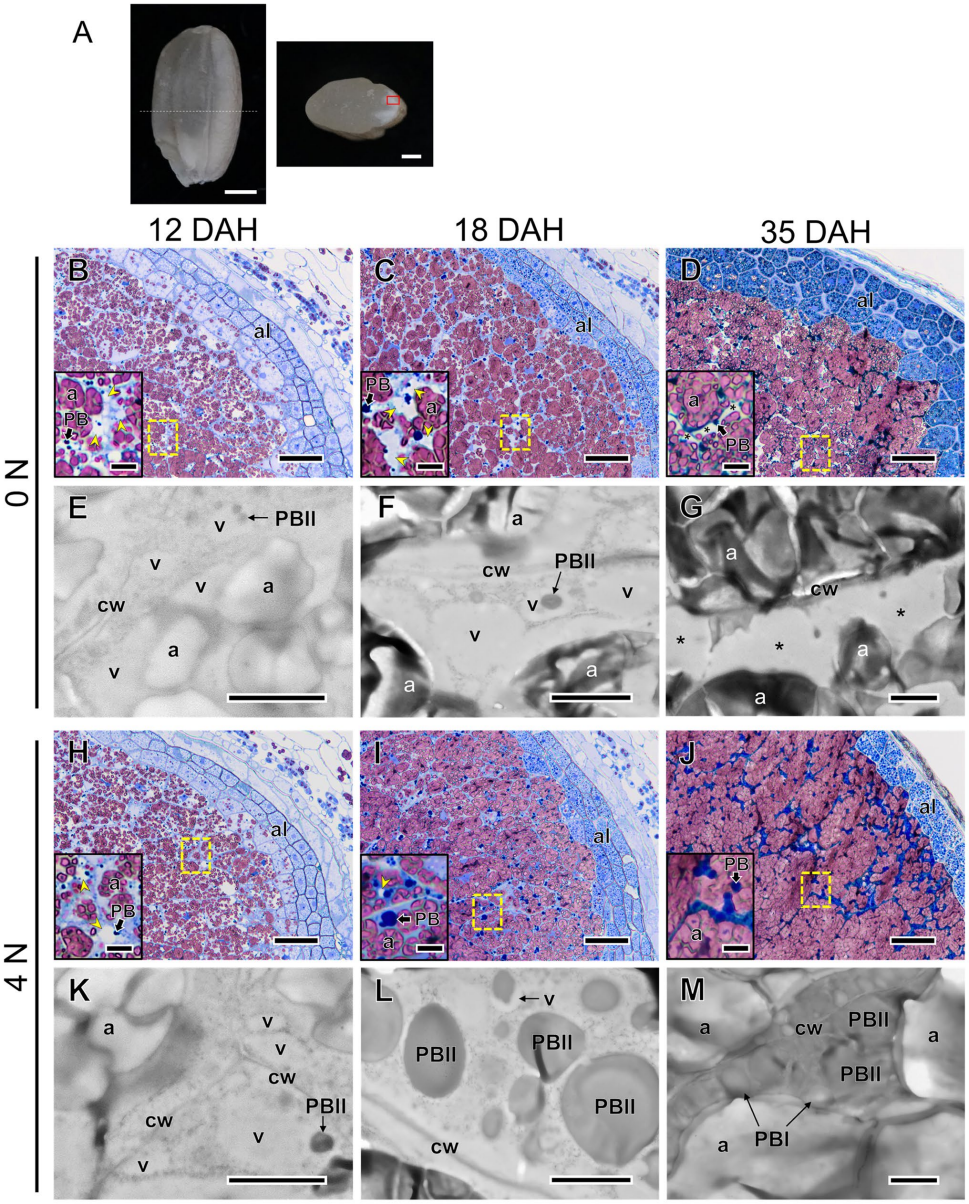
**A** Light microscope image of a typical CK and its chalkiness observable on the cross section of CK at maturation (35 DAH). **B**–**M** Structural and ultrastructural changes of OE cells at the dorsal area of cross sections, corresponding to the rectangle area surrounded with the red dot line shown in **A**, of the kernels in 0 N (**B**–**G**) and 4 N (**H**–**M**) treatments at 12 DAH (**B**, **E**, **H**, **K**), 18 DAH (**C**, **F**,** I**, **L**) and 35 DAH (**D**, **G**, **J**, **M**). **B**–**D**, **H**–**J** Light microscopic images. Each kernel section was double-stained with Coomassie brilliant blue and iodine-potassium iodide. The expanded images of the square areas surrounded by the dashed yellow line were inserted as each inset in the left-bottom corner. Arrowheads in yellow and asterisks in the insets indicate vacuoles and vacuole-like structures, respectively. **E**–**G**, **K**–**M** TEM images. a, amyloplast; al, aleurone layer; PB, protein body. Bars = 1 mm (**A)**, 50 µm (**B**–**D, H–J**), 1 µm (**E**–**G**,**K**–**M**), 10 µm in insets (**B**–**D, H**–**J**)

Panicles (3-4 per block) at 12, 18, and 35 DAH were collected in the field. Immediately, kernels from four superior spikelets attached to the upper position of each panicle were fixed and embedded for microscopic observation, according to previous reports (Saito et al. 2010; Hatakeyama et al. 2018). Semi-thin sections (~ 900 nm) for light microscopy were stained with 0.1% (w/v) Coomassie Brilliant Blue (CBB) for 1 h followed by potassium iodide for 1 min, and ultra-thin sections (80–100 nm) for electron microscopy were stained with lead citrate. After staining, the ultra-thin sections were observed with a TEM (JEM-1010 and JEM 1400 Plus, JEOL Ltd., Tokyo, Japan). For the image analysis of the arrangement of organelles, the outline of all amyloplasts, PBs, vacuoles, and other areas (cytosol including vacuoles, referred to as ‘gaps’) in the cells, as well as cells from light-microscope images taken from three-four plants per treatment, were traced using the ImageJ software (https://imagej.nih.gov/ij/), as described previously (Hatakeyama et al. 2018; Wada et al. 2019). At 12 and 18 DAH, the area of vacuoles (see the arrowheads in Fig. 1B–D, H and I) whose size was larger than 1 μm^2^ was similarly quantified. The areas of amyloplasts, PBs, and vacuole-like structures on TEM images were similarly traced according to Wada et al. (2019). Assuming that these organelles and the cells were spherical, the ratio of volume (V = 4/3πr^3^) to the area (A = πr^2^) was 4/3r, and this value was used to calculate V from A: V = 4/3rA. The spatial ratio of each PB per cell was estimated, and the volume of dorsal OE cells was also calculated with the cell area.

### Protein extraction from rice kernels and SDS-PAGE

SDS-PAGE was conducted using the dorsal side of the mature kernels, corresponding to the chalky zone of WBK and one-third of the total kernel, according to the previous study (Wada et al. 2019). Samples of extracted proteins were separated by SDS-PAGE with 10 − 20% acrylamide and stained with CBB.

### Rice appearance, kernel weight, and dimension

The numbers of the perfect kernel (PK), CK, WBK and BWK, and other immature kernels which had shorter and/or thinner chalky area at the dorsal and basal sides than formal WBK and BWK, respectivey, were evaluated visually for kernels > 1.8 mm in thickness, according to the standard evaluation method of The Ministry of Agriculture, Forestry and Fisheries of Japan (http://www.maff.go.jp/j/seisan/syoryu/kensa/pdf/genmai_kaisetsu.pdf). The data were collected with 113–201 and 341–449 grains pooled from every five plants per block in 2017 and 2018, respectively. The length, width, and thickness of the kernels were also measured using a digital caliper, and the transversal area of the kernels was calculated as an ellipsoid, using 8–18 kernels collected from three independent plants.

### Statistical analysis

For the filled kernels, rice appearance, and kernel protein contents, an analysis of variance (ANOVA) was conducted with the treatments, year, and their interaction as fixed effects and the replications within a year as random effects by using a general linear model (GLM) procedure from JMP (version 13, SAS Institute Inc., Cary, NC, USA). The results of morphological analyses, including the number and area of vacuoles in the OE cells collected at 12, 18, 35 DAH, and cell wall thickness in the dorsal OE cells, the number and areas of each PB per cell, the percentage of PBs in OE cells, and the PBII/PBI ratio between 0 and 4 N treatments were compared using Student’s t-test. Analysis of all other data was conducted using Tukey’s test in JMP.

## Results

### Rice appearance

The daily mean temperatures during the 20 days after heading in 2017 and 2018 were 29.2 °C and 28.3 °C, respectively. The trends of daily mean temperature during the early to middle ripening stage in 2017 were overall greater than in 2018, despite varied sunshine duration and some precipitation (Fig. S1). There were no differences in filled kernels in each year with regard to treatment, suggesting that kernel development similarly occurred in each treatment (Table 1). Table 1 indicates that there was no interaction between treatment and year on the proportion of PK, CK, WBK, and BWK. In 0 N treatment, the proportion of CK was 25.0% on average (Table 1). The N application declined CK (22.0% in 2 N treatment and 10.7% in 4 N treatment), WBK (9.7% in 2 N treatment and 4.3% in 4 N treatment), and BWK (12.3% in 2 N treatment and 6.4% in 4 N treatment), resulting in high proportion of PK (35.8% in 2 N treatment and 50.6% in 4 N treatment) (Table 1). The reduction of the proportion of CK and WBK was much greater in the 4 N treatment than in the 2 N treatment, particularly in 2017 (Table 1). Because of the critical deterioration of rice quality in 2017 mainly due to the occurrence of WBK and other immature kernels under the above-mentioned higher average temperature during the 20 DAH, the following microscopic observations and SDS-PAGE analysis were conducted in 0 N and 4 N treatments in 2017, respectively, to clarify the morphological effects on N application in the corresponding OE cells.

**Table 1:**
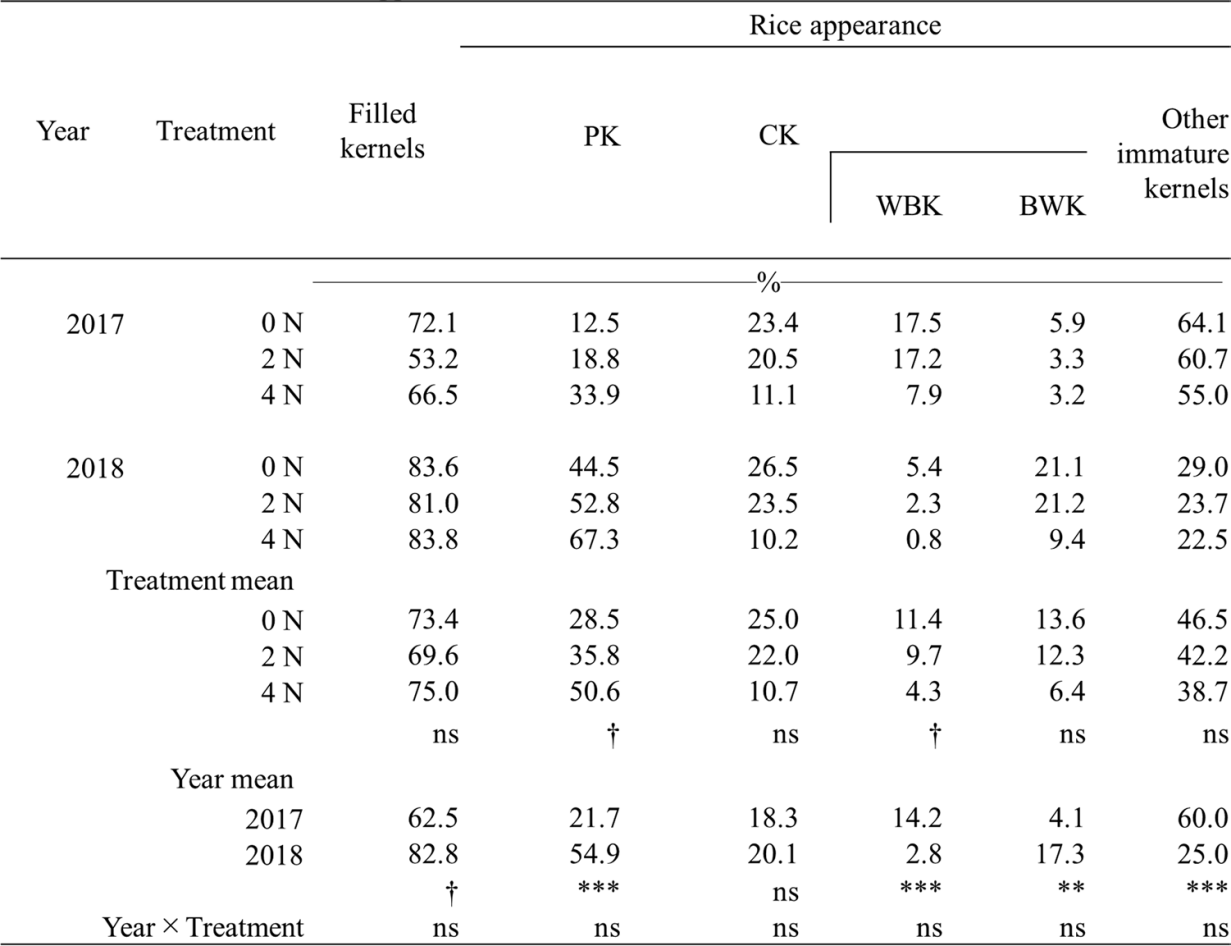
Filled kernels and rice appearance in 0 N, 2 N, and 4 N treatments in 2017 and 2018

ns indicates no significant difference at the 5% level

*** denotes statistical significance at the 0.1% level

^†^ denotes statistical significance at the 1% level

### Microscopic observations

There was no difference in the longitudinal length, width, and thickness of CK and PK in 0 N and 4 N treatments at 35 DAH in 2017 (Table S1). Figure 1 shows the time course of changes in cell morphology and ultrastructure in the OE cells at the dorsal side of the kernels, where the highest frequency of WBKs was recorded at maturation. In light microscopic images, starch granules and PBs (both PBI and PBII) were observed as reddish-purple and blue, respectively, by dual-staining with CBB and iodine-potassium iodide (see Fig. 1B–D, H–J). Although there was no obvious difference in the cell morphology between treatments at 12 DAH (Fig. 1B, E, H, K), numerous larger vacuole-like structures in 0 N treatment than that in 4 N treatment have been observed at 18 DAH (Fig. 1C, F, I, L). When the OE cells at 18 DAH were closely inspected by using TEM, there were many vacuoles (lytic vacuoles and protein storage vacuoles) among amyloplasts in the cells in 0 N treatment (Fig. 1F). Contrastingly in 4 N treatment, few or little vacuoles, but with much larger PBs were observed in the TEM images of 0 N treatment (Fig. 1L). At 35 DAH, many large gap spaces among amyloplasts have been observed in 0 N treatment (Fig. 1D), whereas amyloplasts and PBs were tightly filled in OE cells with small gap spaces in 4 N treatment (Fig. 1J, M). When observing the gap spaces at 35 DAH by using TEM, numerous large vacuole-like structures among the loosely packed amyloplasts and small PBs were preserved in 0 N treatment (Fig. 1G).

**Fig. 2:**
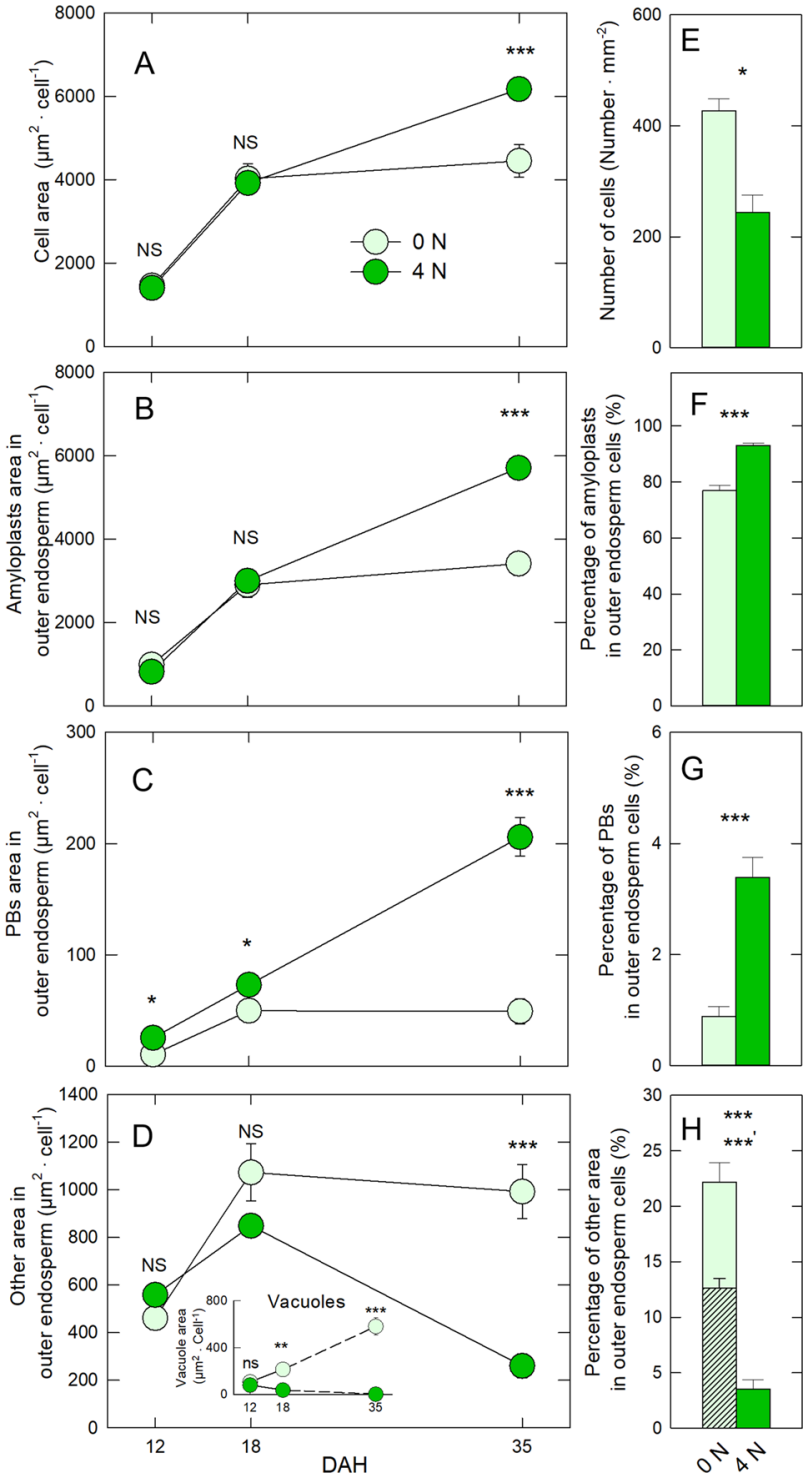
Time-course of changes in the cell areas of OE (**A**) and the within-cell major organelles, amyloplasts (**B**), PBs (**C**), and other areas (gaps/air spaces) (**D**) in 0 N and 4 N treatments during the ripening stage, and the number of the cells (**E**) and the area-based percentages of amyloplasts (**F**), PBs (**G**), and other areas (gaps/air spaces) (**H**) per cell at 35 DAH in each treatment. Inset in **D** indicates changes in the area of vacuoles occupied in the cells during development in each treatment. The hatched area in **H** shows the percentage of the vacuole-like structures that remained per cell at 35 DAH. The data are means (± SE) of 11–15 individual cells collected from three individual plants in each treatment. Significance between treatments at the 0.05 and 0.001 probability levels is indicated with * and ***, respectively, determined using Student’s t test

**Fig. 3:**
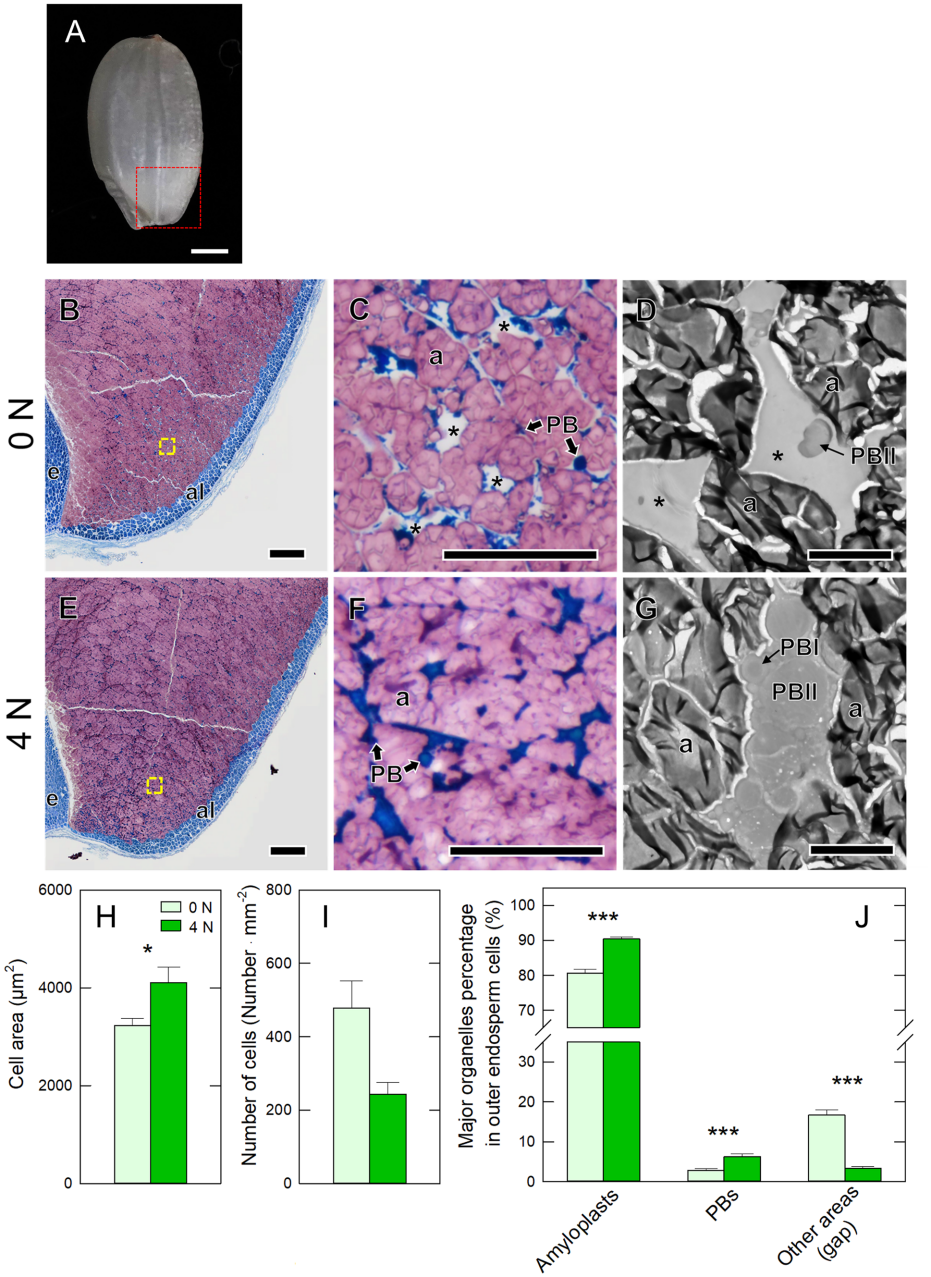
Light microscope image of a typical BWR at maturation (35 DAH) (**A**). **B**–**G** Structural and ultrastructural changes of the endosperm cells at the basal part, corresponding to the rectangle area surrounded by the red dot line shown in **A**. Cell area of the kernels in 0 N (**B**-**D**) and 4 N (**E**–**G**) treatments at 35 DAH. Light microscope images at low (**B**, **E**) and high magnifications (**C**, **F**). TEM images (**D**, **G**) of the same zone in each treatment. Sections in **B** and **E** were double-stained with Coomassie brilliant blue and iodine-potassium iodide. Cell area (**H**), the number of cells per area (**I**), the area-based percentages of amyloplasts, PBs, and other areas (gaps/air spaces) per cell at 35 DAH in each treatment (**J**). The data are means (± SE) of 12–13 individual cells collected from three individual plants in each treatment. Significance between treatments at the 0.001 probability level is indicated with ***. a, amyloplast; al, aleurone layer; e, embryo; PB, protein body. Arrowheads in yellow indicate vacuole-like structures. Bars = 200 µm (**B**, **E**), 50 µm (**C**, **F**), 1 µm (**D**, **G**)

The areas of the cell, amyloplasts, PBs, and vacuoles at 12, 18, and 35 DAH were quantified by light microscopic images (Fig. 2). There were no treatment differences in cell area and amyloplast between 12 and 18 DAH, while the treatment differences were significantly larger in 4 N-treated cells than 0 N-treated cells at 35 DAH (Fig. 2 A and B). By contrast, PBs area in 4 N treatment was larger than that in 0 N treatment throughout the ripening stages (Fig. 2C). The morphological differences in vacuole-like structures clearly differed between treatments during ripening stages. The vacuole area in the 0 N-treated cells gradually increased during ripening stage, whereas the corresponding area in 4 N treatment decreased over time, showing a significant difference in the vacuoles area between treatments at 18 DAH (see the insert in Fig. 2D). At maturation (35 DAH), the area of vacuole-like structures corresponded to more than 50% of the gap area in 0 N treatment, although no vacuole-like structures were observed in 4 N treatment (Fig. 3H). In the mature kernels, the percentage of amyloplasts and PBs in OE cells in 0 N treatment was much lower than in 4 N treatment (Fig. 2F and G). The area of OE cells increased as the number of OE cells declined in 4N treatment (Fig. 2E). Approximately 22% of the other area (i.e., gap/air spaces) predominantly composed of vacuole-like structures was observed in the OE cells in 0 N treatment, whereas the area in 4 N-treated cells was only 4% with no vacuole-like structures (Fig. 2H). There were no treatment differences in cell dimensions at 35 DAH (Table S2), although the cell wall at 35 DAH was prone to be thinner in 0 N treatment than 4 N treatment (Table S2).

**Fig. 4:**
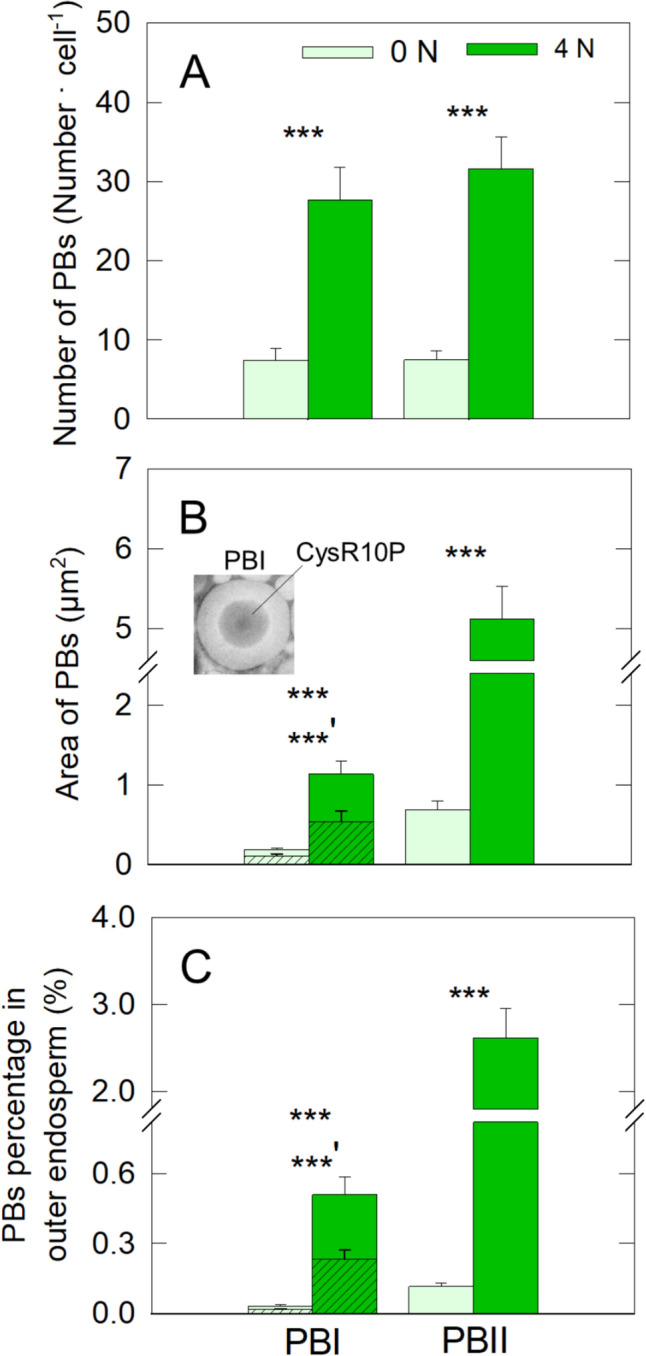
Numbers, areas, and the area-based percentage of PBI and PBII in the OE cells at 35 DAH. The hatched areas of PBI (**B**, **C**) show the Cys-rich 10-kDa layer located at the center of the PBIs. Data are means (± SE) for 12–91 PBs from at least three plants. Significance between treatments at the 0.001 probability level is indicated with ***; significance for the Cys-10-kDa layer with the hatched bars in **B** and **C** is indicated by ’

In addition to the inspection in the dorsal area on cross-section, the basal area of rice kernel has also been observed under light microscope and TEM in terms of formation of another type of heat-induced BWK (Fig. 3). In the chalky zone of BWK in 0 N treatment, numerous large gap spaces were observed in each cell (Fig. 3B and C), whereas such gap spaces were little observed in the corresponding zone of PK in 4 N treatment (Fig. 3E and F). The close inspection indicates that these gaps were composed of many vacuole-like structures formed among loosely packed amyloplasts and PBs (Fig. 3D), contrastingly different from the cell morphology observed in 4 N treatment (Fig. 4G). Unlike 4 N treatment exhibiting normal PB development, the size of PBIIs formed in the vacuole-like structures was found to be arrested and considerably smaller in 0 N treatment (Fig. 3D and G). These patterns on organelles compartmentation observed at the basal area were confirmed to be consistent with those at the dorsal area described above.

### Effects of N treatment on protein body

Image analyses of the areas of PBI and PBII with TEM show that N applications increased the numbers and areas of both PBs (Fig. 4A and B). The ratio of the area of PBII to that of PBI per cell was higher in 4 N treatment than 0 N treatment (Table S3), indicating that N applications led to enhancements of PBII formation and accumulation, compared with PBI. The ratio of the apparent area of CysR10P in the PBIs of 0 N and 4 N treatments was estimated to be 56.6 and 47.9%, respectively (Fig. 4B).

The SDS-PAGE analysis showed that N application promoted the accumulation of PBII-related proteins, pro-glutelin, α-glutelin, α-globulin, and β-glutelin (Fig. S2). In contrast, the content of PBI-related proteins composed of prolamins overall decreased in 4 N treatment, compared with 0 N treatment (Fig. S2). This pattern was consistent with the area-based ratio of PBI and PBII calculated from the results of TEM analysis (Fig. 4C).

## Discussion

These results obtained in field-grown Koshihikari plants indicate that the preservation of vacuole-like structures and the partial arrest of amyloplast development in the OE cells under low N conditions at high temperatures account not only for gap space formation of WBR, but also of BWR. Furthermore, N application reduced the gap space formation by enhancing protein synthesis, even under heat conditions (Figs. 1, 2, 3, 4). Our microscopic observations focusing on organelle development in the OE cells clearly showed that numerous large vacuoles remained at 18 DAH in 0 N treatment (Figs. 1C,F and 2D). In addition, the area of PBs in OE considerably declined but with a progressive increase in vacuolar areas in 0 N treatment, indicating that the protein synthesis would be inhibited in OE cells during maturation (Fig. 2D and G, also see the inset of Fig. 2D). The observed expansion of the vacuolar compartments, mostly cytosolic PSVs with a partial reduction in protein synthesis observed at 18 DAH in 0 N treatment, would be attributed to the poor development of amyloplasts and PBs observable at maturation (Fig. 1B–D), agreeing with the previous work (Wada et al. 2019). In most studies, the effective gap space directly associated with loss of transparency (i.e., formation of chalkiness) has little been evaluated, except by our group. In the present study, it has been demonstrated that the ratio of gap spaces in mature OE cells reached approximately 22% (Fig. 2D), spaces much greater than the threshold that causes significant transparency loss in OE in field-grown plants (see Introduction). On the contrary, adequate N application reduced the vacuolar areas but promoted amyloplast and PB accumulation in OE cells (Figs. 1 and 2). In consequence, the gap space diminished to be ca. 4% below the threshold, increasing transparency in the dorsal OE (Figs. 1H–M and Fig. 2D). Likewise in the basal OE, a similar pattern of organelle compartmentation has been observed (Fig. 3). Hence, these results obtained in the fields demonstrate that preservation of large cytosolic PSVs due to the arrested protein synthesis and inadequate amyloplast accumulation are the main causes of the formation of air spaces in the dorsal and basal OE regions in heat-induced chalky rice, and N-applied OE cells sustained protein synthesis to suppress chalky formation by maintaining PBs and amyloplast development even under heat conditions.

### Impact of vacuolar compartments on chalky formation

After endosperm cell expansion along with vacuole expansion has been completed at normal temperature, vacuolar compartments start to decrease according to the accumulation of amyloplasts and PBs, resulting in the formation of densely packed starchy endosperms (Hoshikawa 1989). Hatakeyama et al. (2018) showed that the preservation of vacuolar structures among loosely packed starch granules in the cytosol of inner endosperms accounts for dry wind-induced air space formation, resulting in ring-shaped chalkiness. Likewise, in WBK, it has been reported that vacuole-like structures remained among the loosely packed starch granules accompanying the inhibition of protein synthesis in the growth chamber experiment (Wada et al. 2019). Therefore, there might be a similarity in the pattern of endosperm organelle compartmentations among the chalky types. In this field experiment, the expansion of vacuoles had likely completed in the putative chalky cells by 18 DAH as none of the cell size and amyloplast areas were different between treatments. And, the enlargement and preservation of vacuoles appeared to be synchronized with the disruption of amyloplast and PB development, which might be responsible for the air space formation in the putative chalky zone in both dorsal and basal regions (Figs. 1, 2, 3, 4). Cell-specific analysis revealed that in the putative chalky cells considerable solute accumulation, mainly sugars and amino acids, might have occurred by osmotic adjustment under heat conditions (Wada et al. 2019). Involvement of osmotic adjustment has been pointed out in various heat-related damages, such as foehn-induced chalky ring formation (Wada et al. 2011, 2014; Hatakeyama et al. 2018), heat-induced chalky formation (Wada et al. 2019), and kernel weight reduction at nighttime warming (Wada et al. 2021). As suggested previously (Wada et al. 2019), the enlargement and preservation of the vacuole-like structures also observed in field-grown plants would be the consequence of osmotic adjustment. In addition, it is reasonably assumed that smaller areas of amyloplasts and PBs (Fig. 2B and C) and thinner cell wall (Table S2) in 0 N treatment than 4 N treatment might result from osmotic adjustment since accumulation of the solutes in cytosol, such as sugar and amino acids should increase the cell osmotic pressure to sustain cell turgor for kernel development (see discussion in Wada et al. 2019). Therefore, it is emphasized that osmotic adjustment should be considered to improve kernel quality under climate change.

### Effects of N application on chalky formation

When considering that starch is the major compound, occupying > 90% of the endosperm cell, it may not be surprising that most studies on heat-induced chalky formation have focused on the regulation of starch synthesis and/or starch degradation in the kernels (Bechtel and Juliano 1980; Hoshikawa 1989). On the other hand, there is accumulating evidence that protein synthesis could play crucial roles on chalky formation in the endosperm cells (Kawakatsu et al. 2010; Tang et al. 2018; Wada et al. 2019; Ishimaru et al. 2020). Furthermore, it has been reported that the occurrence of CK at high temperatures was more sensitive to the amount of nitrogen uptake per spikelet before heading, rather than carbohydrate availability (Tsukaguchi et al. 2011, 2016). The present study showed that the effect of N application for promoting PB accumulation was observed at 12 DAH under the elevated temperature conditions, whereas N-enhanced effect on amyloplast development was observed after 18 DAH (Fig. 2B and C). This indicates that PB development might be more sensitive to N availability, compared with amyloplast development.

It has been reported that adequate N application increased the activities of glutamine synthetase (GS), glutamate synthase (GOGAT), glutamic oxaloacetate transaminase and glutamate pyruvate transaminase but tended to decrease the activities of adenosine diphosphate glucose pyrophosphorylase, granules bound starch synthase, soluble starch synthase, and starch branching enzyme in the kernel at 10 days after anthesis (Fei et al. 2023), consistence with our results. The previous work estimated that the majority of the increase in proteins (~ 65.8%) resulting from N applications would be synthesized as storage proteins in the PBs to fill up the air spaces in the chalky zone (Wada et al. 2019). Given these data, the enhancement of protein synthesis at the early ripening stage might be the prerequisite for ensuring the activity of starch synthesis-related enzymes. Kaneko et al. (2016) reported that ROS-scavenging enzymes might be activated in rice chalky kernels to alleviate such oxidative damage. Considering the involvement of antioxidant metabolites in putative chalky cells (see Wada et al. 2019), the enhanced protein synthesis by N application might prevent the accumulation of excessive ROS through an increase in ROS-scavenging enzymes such as SOD, ascorbate peroxidase, catalase, or glutathione peroxidase. Furthermore, it was reported that N application increased the activities of GS and GOGAT, which are essential to transport the amino acid to OE cells (Wang et al. 2021; Fei et al. 2023), and an accumulation of amino acids in the dorsal OE cells by N application was reported (Wada et al. 2019). Since amino acids could not only be used as material of proteins but also play roles as osmotically active solutes, the enhancement of the supply of amino acids should have large effects on the morphology of vacuoles and PBs in endosperm cells and rice appearance discussed above. Therefore, we propose that maintaining protein biosynthesis in the endosperm cells would be the key to understand the organelle rearrangement in the endosperm cells prior to chalky formation.

### Possible causes of the decrease in PB formation during organelle rearrangement

TEM observation reveals that N application at 20 and 10 days before heading considerably increased the number and area of both PBI and PBII at maturation (Fig. 4A and B). While there was no significant treatment difference in the number of each PB per cell (Fig. 4A), the area of PBII was shown to be five times greater than PBI in the translucent cells (Tabe S3). Some researchers suggested that the prolamin synthesis might have an impact on generating the chalkiness and improvement of rice appearance by the N application (Yamakawa et al. 2007; Ishimaru et al. 2019, 2020; Wang et al. 2021), however, there was no spatial analysis conducted microscopically in terms of chalky formation in the cells. Our TEM-based site-specific analysis showed that an increase in the PBII/PBI ratio occurred by N application, resulting in the improvement of rice appearance (Table S3), suggesting that PBII development would be more sensitive as that of PBI under N-enhanced field conditions. Our previous study indicated that N-enhanced PBIIs would fill up the matrix of PSV as the main cause of air space in the OE cells (Wada et al. 2019). Most rice mutants that suppressed genes of processes of transport of PBII-related proteins to PSVs by Golgi typically exhibit chalky appearance as a morphological trait (Fukuda et al. 2011; Ren et al. 2014). Therefore, it is strongly suggested that PBII development would be crucial for rice appearance under heat conditions and the synthesis of glutelin and globulin might play a critical role in rice appearance throughout the volumetric regulation of PBII, rather than that of PBI.

Regarding the PBI development, the pattern for the ratio of area of CysR10P per PBI in response to N supply at high temperature did differ among studies (Table S3, Fig. S1 and see Wada et al. 2019). These differences might be attributed to the differences in the timing of N application and the extent of heat conditions, as well as the difference in sink-to-source ratio and light conditions between the cultivations. Recently, single-cell metabolomics revealed that greater Cys content was frequently observed in the heat-treated OE cells, but not for control and N-applied heat treatment (Wada et al. 2019). In the present study, single-cell metabolomics was not conducted; however, the results are suggestive that the same mechanisms likely exist in the OE cells in the field-grown plants. If this were the case, the formation of disulfide bonds with Cys would increase in the N-applied cells during the heat acclimation process in the fields. Interestingly, most of the rice mutants that suppressed genes encoding proteins involved in the formation of disulfide bonds (Takemoto et al. 2002; Onda et al. 2011; Han et al. 2012) show chalkiness. It is anticipated that insufficient N supply under heat conditions in the fields would reduce the accumulation of storage proteins with disulfide bonds, resulting in the deteriorated rice appearance. Further studies will be required to determine the effect of high temperature on the formation of disulfide bonds in terms of rice quality.

In addition, cell morphology for chalky and translucent endosperm cells located at the basal part of kernels were found to be similar to those at the dorsal part; (i) there were numerous large vacuole-like structures among amyloplasts, and (ii) amyloplasts and PBs areas in chalky cells were smaller than those in translucent cells (Fig. 3). Similar to the dorsal chalkiness (i.e., WBK) discussed above, preservation of vacuoles with arrested protein synthesis might account for the basal chalky formation. Our findings suggest that heat-induced chalky formation is directly caused by the preservation of vacuoles and the disruption of protein biosynthesis accompanied by a reduction in starch accumulation. It has also been shown that N application could enhance protein synthesis, which improves rice quality in field-grown rice plants exposed to heat.

## Conclusion

In this study, we hypothesized that rice chalkiness in heat-exposed field-grown plants is attributed to the preservation of vacuolar compartments due to the arrested protein synthesis in endosperm cells, depending on the N availability. By using light microscopy and TEM, the pattern of organelle compartments in both dorsal and basal endosperms was examined with or without N supply. When the N level was insufficient under heat, the OE cells exhibited a large proportion of enlarged vacuolar compartments originated from PSV, accompanied by a small proportion of starch granules and PBs in the cytosol, leading to chalky kernels. In contrast, N-enhanced plants showed normal amyloplast and protein body (namely PBII) development, diminishing the vacuolar compartments even under heat conditions. These data indicate that under low-N and high-temperature conditions the preservation of vacuole-like structures and partial arrest of amyloplast development in OE cells leads to the gap space formation of both WBR and BWR. Additionally, N application mitigated gap space formation by enhancing protein synthesis, even under heat conditions. Hence, we conclude that vacuole-like structures preserved among loosely-packed amyloplasts in the endosperm cells are the main cause of heat-induced rice chalky formation, particularly promoted under low N conditions. This underscores that the regulation of vacuolar compartmentation and protein synthesis during the ripening stage plays a crucial role in determining rice appearance under high temperature conditions.

## Supplementary Information

Below is the link to the electronic supplementary material.

Supplementary file1 (PDF 543 KB)

## Data Availability

Data will be available upon request.

## Abbreviations

BWK: Basal-white kernel
CK: Chalky kernel (White-back and basal-white kernels)
CysR10P: Cysteine-rich 10-kDa prolamin
DAH: Days after heading
OE: Outer endosperm
PB: Protein body
PSV: Protein storage vacuole
TEM: Transmission electron microscopy
WBK: White-back kernel
