# Supplementary material for: Vacuolar compartments preserved among loosely packed amyloplasts account for heat-induced rice chalky formation under low nitrogen conditions
Source: Planta. 2025 Aug 13;262(4):79. doi: 10.1007/s00425-025-04793-z (PMC12350539; doi:10.1007/s00425-025-04793-z)
Supplement: Supplementary file 1 — Supplementary file1 (PDF 543 KB) [file 425_2025_4793_MOESM1_ESM.pdf]

Supplementary Information

Vacuolar compartments preserved among loosely-packed amyloplasts account for heat-induced rice chalky formation under low nitrogen conditions

Yuto Hatakeyama[1,2*], Kenichi Wakamatsu[3], Akio Tanaka[3], Taku Tanogashira[3], Hiroshi Nonami[2], Hiroshi Nakano[1], and Hiroshi Wada[1,2*]

[1] Kyushu Okinawa Agricultural Research Center, National Agriculture and Food Research Organization, Chikugo, Fukuoka, Japan
[2] Graduate School of Agriculture, Ehime University, Matsuyama, Ehime, Japan
[3] Kagoshima Prefectural Institute for Agricultural Development, Minamisatsuma, Kagoshima, Japan.

* Authors for correspondence: Yuto Hatakeyama and Hiroshi Wada;
Emails: hatakey@agr.ehime-u.ac.jp, hwada@agr.ehime-u.ac.jp

Table S1. The longitudinal length, width, and thickness of CK in 0 N treatment and PK in 4 N treatment at 35 DAH.

Treatment	Length	Width	Thickness
	mm		
0 N	5.36	3.05	2.03
4 N	5.29	2.91	2.04
Treatment effect	ns	ns	ns

ns, no significance at the 0.05 probability level.

Table S2. Cell wall thickness of the dorsal outer endosperm cells in 0 N and 4 N treatments at 12 and 35 DAH.

Treatment	12 DAH	35 DAH
	——— μm ———	
0 N	0.015	0.079
4 N	0.016	0.163
Treatment effect	ns	***

***, Significance at the 0.001 probability level.
ns, no significance at the 0.05 probability level.

Table S3. The area percentage of PBI and PBII per dorsal outer cells and PBII/PBI ratio in CK in 0 N treatment and PK in 4 N treatment at 35 DAH.

Treatment	Areas of each PB per cell		PBII/PBI ratio
	PBI	PBII	
	—— % ——		$\mu m^2\ \mu m^{-2}$
0 N	0.032	0.115	3.6
4 N	0.508	2.619	5.2
Treatment effect	***	***	ns

***, Significance at the 0.001 probability level.
ns, no significance at the 0.05 probability level.

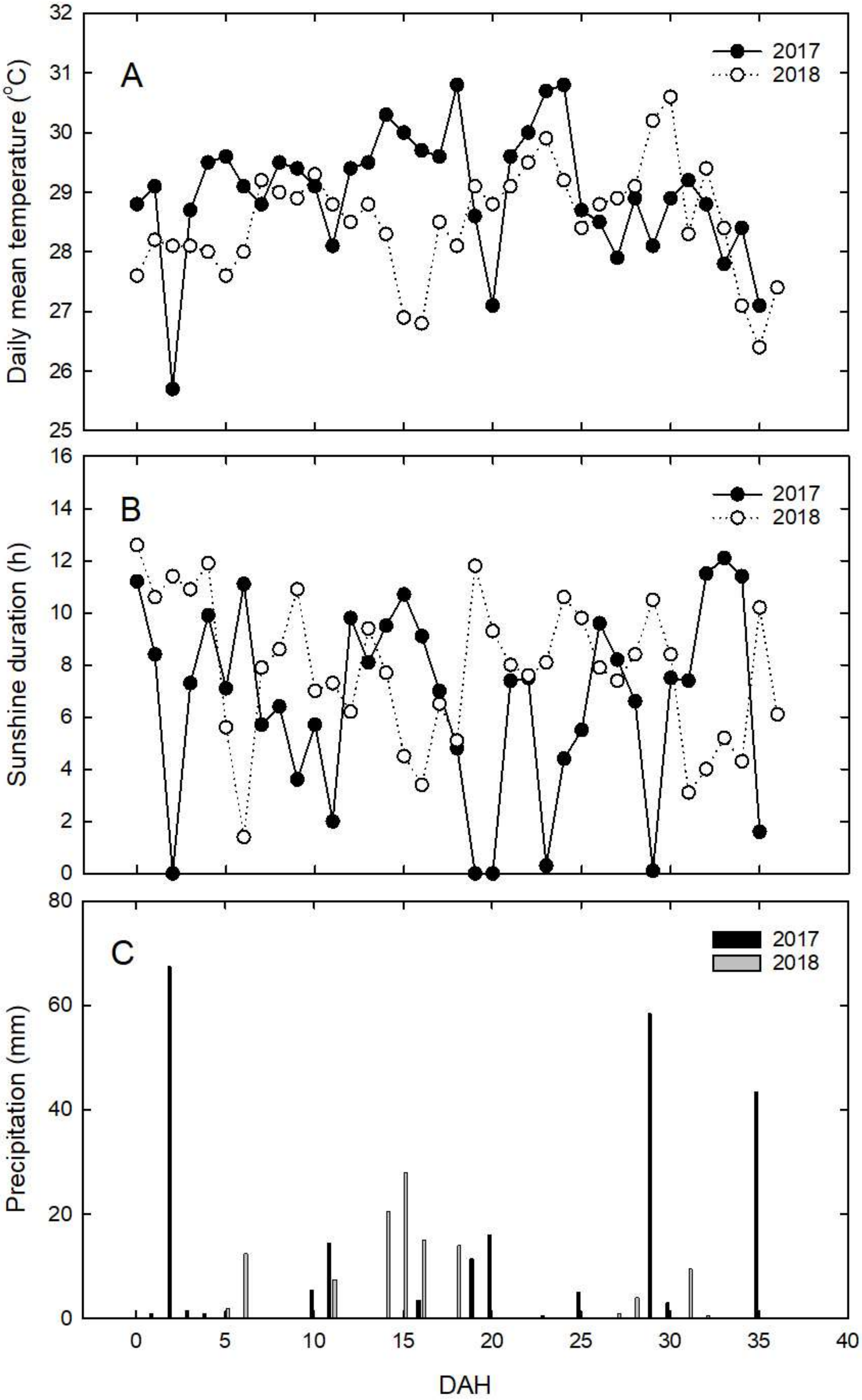

Fig. S1. Changes in mean air temperature (A), sunshine duration (B), and precipitation (C) throughout the ripening stage in 2017 and 2018.

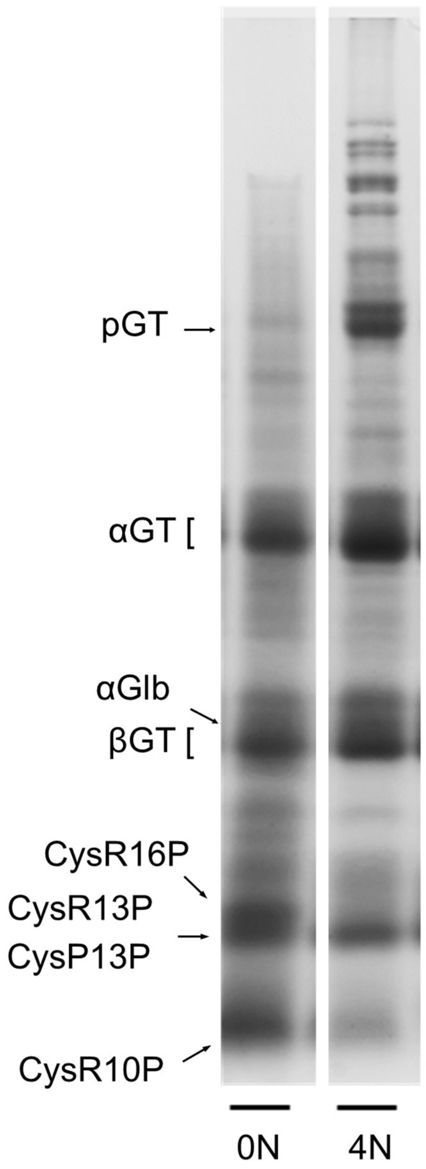

Fig. S2. SDS-PAGE analysis of the dorsal endosperm tissues of CK in 0 N treatment (left) and PK in 4 N treatment (right). CysR10P, Cys-rich 10-kDa prolamin; CysP13P, Cys-poor 13-kDa prolamin; CysR13P, Cys-rich 13-kDa prolamin; CysR16P, Cys-rich 16-kDa prolamin; βGT, glutelin basic subunit; αGlb, α-globulin; αGT, glutelin acidic subunit; pGT, proglutelin.
